# Supplementary material for: Impaired estimated glomerular filtration rate and associated factors among adult patients living with HIV at Asella Referral and Teaching Hospital, Ethiopia: A cross-sectional study
Source: PLoS One. 2026 Jan 2;21(1):e0330238. doi: 10.1371/journal.pone.0330238 (PMC12758773; doi:10.1371/journal.pone.0330238)
Supplement: S1 File — S1 Impaired eGFR Data Collection Tool. S2 Impaired eGFR among HIV source data file. S3 Table 5 Bivariate analysis of factors associated with impaired glomerular filtration rate. S4 Table 6 Multivariable analysis of factors associated with impaired estimated glomerular filtration rate. (ZIP) [file pone.0330238.s001.zip › S3-Table 5 Bivariate analysis of factors associated with impaired glomerular filtration rate among adults living with HIV at Asella Referral and Teaching Hospital (N=252)..docx]

Table 5: Bivariate analysis of factors associated with impaired glomerular filtration rate among adults living with HIV at Asella Referral and Teaching Hospital (N=252).

| **Variables** | **Impaired eGFR (n=47)** | **Normal eGFR (n=205)** | **COR (95% CI)** | **P-value** |
| --- | --- | --- | --- | --- |
| Age (years) |  |  |  |  |
| >=40 | 39 (83.0%) | 129 (62.9%) | 2.87 (1.27–6.46) | 0.011 |
| <40 | 8 (17.0%) | 76 (37.1%) | 1 [Ref] | — |
| **Sex** |  |  |  |  |
| Male | 18 (38.3%) | 96 (46.8%) | 0.70 (0.36–1.34) | 0.29 |
| Female | 29 (61.7%) | 109 (53.2%) | 1 [Ref] | — |
| **Marital status** |  |  |  |  |
| Single | 6 (12.8%) | 42 (20.5%) | 0.66 (0.19–2.27) | 0.51 |
| Married | 27 (57.4%) | 104 (50.7%) | 1.21 (0.45–3.22) | 0.70 |
| Divorced | 8 (17.0%) | 31 (15.1%) | 1.20 (0.37–3.90) | 0.75 |
| Widowed | 6 (12.8%) | 28 (13.7%) | 1 [Ref] | — |
| **Educational status** |  |  |  |  |
| No formal education | 7 (14.9%) | 25 (12.2%) | 0.98 (0.35–2.67) | 0.96 |
| Primary education | 11 (23.4%) | 61 (29.8%) | 0.63 (0.27–1.47) | 0.28 |
| Secondary education | 13 (27.7%) | 63 (30.7%) | 0.72 (0.32–1.63) | 0.43 |
| College and above | 16 (34.0%) | 56 (27.3%) | 1 [Ref] | — |
| **Occupation** |  |  |  |  |
| Unemployed | 10 (21.3%) | 42 (20.5%) | 1.29 (0.34–4.81) | 0.70 |
| Government employee | 9 (19.1%) | 41 (20.0%) | 1.40 (0.37–5.31) | 0.61 |
| Self-employed | 20 (42.6%) | 101 (49.3%) | 1.55 (0.45–5.25) | 0.47 |
| Student | 4 (8.5%) | 8 (3.9%) | 0.62 (0.11–3.17) | 0.56 |
| Others* | 4 (8.5%) | 13 (6.3%) | 1 [Ref] | — |
| **Residency** |  |  |  |  |
| Urban | 33 (70.2%) | 157 (76.6%) | 0.72 (0.35–1.45) | 0.36 |
| Rural | 14 (29.8%) | 48 (23.4%) | 1 [Ref] | — |
| **Income (ETB)** |  |  |  |  |
| <3500 | 22 (46.8%) | 99 (48.3%) | 0.94 (0.49–1.77) | 0.85 |
| >=3500 | 25 (53.2%) | 106 (51.7%) | 1 [Ref] | — |
| **Family history of renal disease** |  |  |  |  |
| Yes | 2 (4.3%) | 5 (2.4%) | 1.77 (0.33–9.45) | 0.50 |
| No | 45 (95.7%) | 200 (97.6%) | 1 [Ref] | — |
| **History of alcohol drinking** |  |  |  |  |
| Yes | 11 (23.4%) | 44 (21.5%) | 1.11 (0.52–2.37) | 0.77 |
| No | 36 (76.6%) | 161 (78.5%) | 1 [Ref] | — |
| **BMI** |  |  |  |  |
| Underweight | 16 (34.0%) | 41 (20.0%) | 2.26 (1.09–4.65) | 0.042 |
| Overweight | 7 (14.9%) | 25 (12.2%) | 1.62 (0.63–4.16) | 0.31 |
| Normal | 24 (51.1%) | 139 (67.8%) | 1 [Ref] | — |
| **History of smoking** |  |  |  |  |
| Yes | 16 (34.0%) | 24 (11.7%) | 3.89 (1.86–8.14) | <0.001 |
| No | 31 (66.0%) | 181 (88.3%) | 1 [Ref] | — |
| **CD4 count (cells/mm³)** |  |  |  |  |
| <200 | 10 (21.3%) | 12 (5.9%) | 4.34 (1.75–10.79) | 0.002 |
| >=200 | 37 (78.7%) | 193 (94.1%) | 1 [Ref] | — |
| Variables | Impaired GFR (n=47) | Normal GFR (n=205) | COR (95% CI) | P-value |
| **ART interruption history** |  |  |  |  |
| Yes | 17 (36.2%) | 39 (19.0%) | 2.41 (1.21–4.80) | 0.012 |
| No | 30 (63.8%) | 166 (81.0%) | 1 [Ref] | — |
| **Opportunistic infections** |  |  |  |  |
| Yes | 26 (55.3%) | 48 (23.4%) | 4.05 (2.07–8.03) | <0.001 |
| No | 21 (44.7%) | 157 (76.6%) | 1 [Ref] | — |
| **Diabetes mellitus (DM)** |  |  |  |  |
| Yes | 14 (29.8%) | 26 (12.7%) | 2.92 (1.38–6.17) | 0.005 |
| No | 33 (70.2%) | 179 (87.3%) | 1 [Ref] | — |
| **Hypertension** |  |  |  |  |
| Yes | 14 (29.8%) | 32 (15.6%) | 2.29 (1.10–4.76) | 0.026 |
| No | 33 (70.2%) | 173 (84.4%) | 1 [Ref] | — |
| **Tenofovir-based regimen** |  |  |  |  |
| Yes | 28 (59.6%) | 152 (74.1%) | 0.51 (0.26–0.99) | 0.048 |
| No | 19 (40.4%) | 53 (25.9%) | 1 [Ref] | — |
| **WHO clinical stage** |  |  |  |  |
| Stage 1 | 18 (38.3%) | 91 (44.4%) | 1 [Ref] | — |
| Stage 2 | 12 (25.5%) | 88 (42.9%) | 0.68 (0.31–1.51) | 0.35 |
| Stage 3 & 4 | 17 (36.2%) | 26 (12.7%) | 3.30 (1.49–7.30) | 0.003 |
| **ART regimen category** |  |  |  |  |
| First line | 37 (78.7%) | 185 (90.2%) | 1 [Ref] | — |
| Second & Third line | 10 (21.3%) | 20 (9.8%) | 0.40 (0.17–0.92) | 0.032 |
| **ART dose frequency** |  |  |  |  |
| Once daily | 40 (85.1%) | 191 (93.2%) | 1 [Ref] | — |
| ≥2 times daily | 7 (14.9%) | 14 (6.8%) | 2.38 (0.90–6.29) | 0.078 |
