# Supplementary material for: Impaired estimated glomerular filtration rate and associated factors among adult patients living with HIV at Asella Referral and Teaching Hospital, Ethiopia: A cross-sectional study
Source: PLoS One. 2026 Jan 2;21(1):e0330238. doi: 10.1371/journal.pone.0330238 (PMC12758773; doi:10.1371/journal.pone.0330238)
Supplement: S1 File — S1 Impaired eGFR Data Collection Tool. S2 Impaired eGFR among HIV source data file. S3 Table 5 Bivariate analysis of factors associated with impaired glomerular filtration rate. S4 Table 6 Multivariable analysis of factors associated with impaired estimated glomerular filtration rate. (ZIP) [file pone.0330238.s001.zip › S4-Table 6 Multivariable analysis of factors associated with impaired estimated glomerular filtration rate among HIV-positive adults at Asella Referral and Teaching.docx]

**Table 6: Multivariable analysis of factors associated with impaired estimated glomerular filtration rate among HIV-positive adults at Asella Referral and Teaching Hospital, Ethiopia (N=252).**

| **Variables** | **Estimated GFR Status n (%)** | | **Crude OR (95% CI)** | **Adjusted OR (95% CI)** | **P-value** |
| --- | --- | --- | --- | --- | --- |
|  | Impaired (n=47) | Normal (n=205) |  |  |  |
| **Age** |  |  |  |  |  |
| 18–39 years | 8 (17.0%) | 76 (37.1%) | 1 (Ref) | 1 (Ref) |  |
| >40–65 years | 39 (83.0%) | 129 (62.9%) | 2.87 (1.27–6.46) | 3.26 (1.17–9.12) | **0.024*** |
| **History of smoking** |  |  |  |  |  |
| Yes | 16 (34.0%) | 24 (11.7%) | 3.89 (1.86–8.14) | 4.68 (1.87–11.70) | **0.001*** |
| No | 31 (66.0%) | 181 (88.3%) | 1 (Ref) | 1 (Ref) |  |
| **CD4 count** |  |  |  |  |  |
| <200 cells/mm³ | 10 (21.3%) | 12 (5.9%) | 4.34 (1.75–10.79) | 3.49 (0.71–17.14) | 0.124 |
| ≥200 cells/mm³ | 37 (78.7%) | 193 (94.1%) | 1 () | 1 (Ref) |  |
| **ART interruption history** |  |  |  |  |  |
| Yes | 17 (36.2%) | 39 (19.0%) | 2.41 (1.21–4.80) | 0.65 (0.20–2.05) | 0.463 |
| No | 30 (63.8%) | 166 (81.0%) | 1 (Reference) | 1 (Ref) |  |
| **Opportunistic infections** |  |  |  |  |  |
| Yes | 26 (55.3%) | 48 (23.4%) | 4.05 (2.07–8.30) | 5.93 (2.23–15.74) | **<0.001*** |
| No | 21 (44.7%) | 157 (76.6%) | 1 (Reference) | 1 (Ref) |  |
| **Diabetes mellitus (DM)** |  |  |  |  |  |
| Yes | 14 (29.8%) | 26 (12.7%) | 2.92 (1.38–6.17) | 3.86 (1.47–10.12) | **0.006*** |
| No | 33 (70.2%) | 179 (87.3%) | 1 (Reference) | 1 (Ref) |  |
| **Hypertension** |  |  |  |  |  |
| Yes | 14 (29.8%) | 32 (15.6%) | 2.29 (1.10–4.76) | 2.71 (1.07–6.82) | **0.034*** |
| No | 33 (70.2%) | 173 (84.4%) | 1 (Reference) | 1 (Ref) |  |
| **Tenofovir-based regimen** |  |  |  |  |  |
| Yes | 28 (59.6%) | 152 (74.1%) | 0.51 (0.26–0.99) | 0.61 (0.24–1.51) | 0.287 |
| No | 19 (40.4%) | 53 (25.9%) | 1 (Reference) | 1 (Ref) |  |
| **WHO clinical stage** |  |  |  |  |  |
| Stage 1 | 18 (38.3%) | 91 (44.4%) | 1 (Reference) | 1 (Ref) |  |
| Stage 2 | 12 (25.5%) | 88 (42.9%) | 0.68 (0.31–1.51) | 0.73 (0.27–1.97) | 0.538 |
| Stage 3 & 4 | 17 (36.2%) | 26 (12.7%) | 3.30 (1.49–7.30) | 0.63 (0.15–2.55) | 0.519 |
| **ART regimen category** |  |  |  |  |  |
| First line | 37 (78.7%) | 185 (90.2%) | 1 (Reference) | 1 (Ref) |  |
| Second & Third line | 10 (21.3%) | 20 (9.8%) | 0.40 (0.17–0.92) | 1.87 (0.30–11.58) | 0.499 |
| **ART dose frequency** |  |  |  |  |  |
| Once daily | 40 (85.1%) | 191 (93.2%) | 1 (Reference) | 1 (Ref) |  |
| ≥2 times daily | 7 (14.9%) | 14 (6.8%) | 2.38 (0.90–6.29) | 0.76 (0.10–5.40) | 0.787 |
