# Supplementary material for: Impaired estimated glomerular filtration rate and associated factors among adult patients living with HIV at Asella Referral and Teaching Hospital, Ethiopia: A cross-sectional study
Source: PLoS One. 2026 Jan 2;21(1):e0330238. doi: 10.1371/journal.pone.0330238 (PMC12758773; doi:10.1371/journal.pone.0330238)
Supplement: Attachment — Submitted filename: Response to reviewers.docx [file pone.0330238.s003.docx]

**Tamiru Adugna, MD**

Assistant Professor of Internal Medicine

College of Health Sciences, Arsi University

Asella, Ethiopia

Email: [adutamiru@gmail.com](mailto:adutamiru@gmail.com)

Mobile: +251-922-710-378

ORCID: <https://orcid.org/0009-0001-6586-8084>

October, 2025

Donovan Anthony McGrowder, PhD, MA, MSc

Academic Editor

PLOS ONE

Subject: Revised Manuscript Submission PONE-D-25-40706

Title: Impaired Estimated Glomerular Filtration Rate and Associated Factors among Adult Patients Living with HIV at Asella Referral and Teaching Hospital, Ethiopia: A Cross-Sectional Study

Dear Dr. McGrowder,

We extend our sincere gratitude to you and the reviewers for the constructive and insightful feedback provided on our manuscript (**ID: PONE-D-25-40706**). The time and effort dedicated to reviewing our work are greatly appreciated, and the comments have been instrumental in enhancing the quality and clarity of the manuscript.

We have thoroughly considered all the points raised and have implemented comprehensive revisions to address each concern. Our detailed, point-by-point responses to the reviewers’ comments are provided below.

Additionally, we have carefully rectified the editorial and formatting inconsistencies that arose during the initial file conversion process. The revised manuscript now ensures clear and accurate presentation of all text, tables, and figures.

**Point-by-Point Responses to Reviewers' Comments**

**Reviewer #1**

**Comment 1:** "People living with HIV (PLHIV) demonstrate diverse renal pathology, including acute kidney injury (AKI), HIV-associated nephropathy (HIVAN), chronic kidney disease (CKD), and antiretroviral therapy (ART)-induced nephrotoxicity. Therefore, what would likely interest the readers of this paper, and what holds clinical significance, is an elucidation of the distinct characteristics associated with each underlying cause of renal impairment. This perspective, however, is lacking in the current manuscript, which I consider to be its primary limitation."

**Response:** We thank the reviewer for this insightful and clinically relevant observation. We fully agree that distinguishing between the various etiologies of renal impairment is crucial for both clinical management and scientific understanding. However, due to the cross-sectional design of our study, which relied on a single serum creatinine measurement, we were unable to definitively differentiate between specific renal pathologies such as AKI, CKD, or HIVAN, as these require longitudinal data or renal biopsy for confirmation. We have explicitly acknowledged this limitation in the revised manuscript.

**Location in the manuscript:** Page 21–22, Lines 329–335

**Comment 2**: Technical suggestions regarding tables.

**Response:** We appreciate the reviewer’s technical suggestions. In response, we have merged Tables 5 and 6 into a single consolidated table to improve clarity. The structure of the remaining tables has been retained to ensure they remain comprehensive yet accessible to a broad readership.

**Location in the manuscript**: Page 17–18, Lines 264–267

**Comment 3**: Technical suggestion regarding figures.

**Response**: We thank the reviewer for the valuable feedback on the figures. Following re-evaluation, we have removed Figure 1 to streamline the presentation. The remaining figures have been retained to support the narrative and enhance visual clarity.

**Reviewer #2**

We express our sincere appreciation to the reviewer for the time and constructive comments. Below are our responses to the specific questions raised:

**Question 1**: What is the average duration of antiretroviral therapy among participants, and how does it influence renal function?

**Response**: The present study did not assess the average duration of ART exposure or its potential impact on renal function. This limitation has been explicitly stated in the revised manuscript to provide clarity for readers.

**Location in the manuscript**: Page 21, Lines 324-327.

**Question 2**: Are there differences between the treatment regimens used?

**Response**: In this cohort, over 88% of participants were on first-line ART, with the majority (71.4%) receiving a TDF-based regimen. No statistically significant association was found between the type of ART regimen and impaired eGFR. This point has been clarified in the revised manuscript.

**Location in the manuscript**: Page 21, Lines 325–328

**Question 3**: Based on these results, could a follow-up evaluation algorithm be developed?

**Response**: As this is a single-center, institution-based study, the findings are not sufficient to establish a follow-up evaluation algorithm. We recommend further multicenter studies to develop and validate such an algorithm.

We are confident that the revisions have strengthened the manuscript and enhanced its clarity and impact. We believe the revised version now meets the high standards of PLOS ONE and hope it is found suitable for publication.

Sincerely,

Tamiru Adugna Dadi, MD

Corresponding Author

Assistant Professor of Internal Medicine

Arsi University, College of Health Sciences
